# Internet chemotherapy information: impact on patients and health professionals

**DOI:** 10.1038/bjc.2011.601

**Published:** 2012-01-19

**Authors:** E Davies, K-W Yeoh

**Affiliations:** 1 Department of Oncology/Haematology, Northampton General Hospital NHS Trust, Cliftonville, Northampton, NN1 5BD, UK; 2 Department of Clinical Oncology, The Churchill Hospital, Oxford Radcliffe Hospitals NHS Trust, Old Road, Oxford OX3 7LJ, UK

**Keywords:** internet, chemotherapy, education of patients, physicians, nurses, health personnel

## Abstract

**Background:**

Reliable information can improve patients’ knowledge of chemotherapy. As internet chemotherapy information (ICI) is increasingly viewed as a valuable patient education tool, we investigated the impact of ICI on patient care and analysed health professionals’ (HPs’) attitudes towards ICI.

**Methods:**

The following questionnaires were distributed: (1) self-administered questionnaire randomly given to 261 patients receiving chemotherapy (80% returned); and (2) separate questionnaire given to 58 HPs at the same UK Oncology Centre (83% returned).

**Results:**

Just over half of the patient respondents accessed the internet regularly. They were younger, with higher incomes and qualifications. Key search topics included chemotherapy modes of action, symptom management and treatment success, and most considered ICI useful. More than half wanted to discuss ICI with HPs but most did not get the opportunity. Although the majority of HP respondents supported the need for patients to retrieve ICI, most questioned the accuracy of ICI and did not routinely recommend its use.

**Conclusion:**

This study has shown that ICI is generally perceived by patients to be a valuable information resource. Given the potential impact of ICI, the following should be addressed in future studies: (1) inequalities in accessing ICI; (2) maintaining the quality of ICI (with clear guidance on recommended websites); (3) bridging the gap between the perception of ICI by patients and HPs; (4) integration of ICI with traditional consultation models.

Patients with cancer primarily obtain information about their condition and treatment from health professionals (HPs). As patient concerns may not always be met by HPs, it is not uncommon for patients to seek information that will improve their understanding of their disease and treatment from other sources, including the internet (Fallowfield *et al*, 1994; Meredith *et al*, 1996; Jenkins *et al*, 2001; Mayer *et al*, 2007).

Access to the internet varies between regions and countries. At the time of this study, approximately 76% of the UK population accessed the internet, with millions among them searching daily for health-related information (Internet Access from the Office of National Statistics, 2011). It has been shown that cancer patients seek online information disproportionately more frequently than other health-related searchers (Chen and Siu, 2001; Pautler *et al*, 2001; Fogel *et al*, 2002; Smith *et al*, 2003; Basch *et al*, 2004; Newnham *et al*, 2006; van de Poll-Franse and van Eenbergen, 2008). With cancer information on the internet being viewed as an increasingly important patient education tool, there may be potential issues with the credibility and quality of websites and access thereto. In the same vein, HPs’ attitude towards cancer information on the internet is increasingly relevant in determining how internet information can be integrated within traditional consultation models, if at all.

Although much work has been based on internet and cancer in general (Biermann *et al*, 1999; Chen and Siu, 2001; Eysenbach, 2003; Helft *et al*, 2003; Helft *et al*, 2005; Newnham *et al*, 2005; Newnham *et al*, 2006; van de Poll-Franse and van Eenbergen, 2008), there have been no studies examining the experience by patients on chemotherapy and by their respective HPs with internet chemotherapy information (ICI). Consequently, the effect of ICI on patient care and outcomes remains largely unknown on this group of patients. The perception of HPs towards ICI is also unknown. This group of patients will potentially be more at risk of experiencing toxicities from cancer treatment and should also have the opportunity for regular interaction with HPs during clinical reviews before ongoing cycles of chemotherapy.

This study investigated the proportion and characteristics of patients who specifically sought ICI while receiving chemotherapy treatment for a variety of tumour types at a cancer centre in the UK. The main aims of the study were to investigate the following: (1) whether ICI was considered beneficial or detrimental by cancer patients and their HPs; (2) the attitudes of HPs to patients who received ICI; and (3) HPs’ awareness of their patients’ needs for information outside the clinical setting, including ICI. The outcomes of this study will be considered in order to determine how HPs may utilise ICI as a meaningful tool to address patient educational needs and for future studies. This paper should also prove to be a useful case study or illustration for other cancer centres.

## Subjects and Methods

### Patients and HPs

This study focused on a group of patients who commenced chemotherapy treatment during a 6-month period and the HP team who were directly involved in their care at the Northamptonshire Oncology Centre (the ‘Centre’), which is based in a 650 bed district general hospital.

Patients were selected by simple random sampling, whereby every third patient attending the day case chemotherapy centre for the second cycle of chemotherapy was given a self-administered questionnaire, during a 6-month period. The cancer types shown in Table 1 represent the most common cancers seen at our centre. Patient inclusion criteria were as follows: (1) age ⩾18 years; (2) able to speak and understand English; and (3) undergoing chemotherapy with a cancer diagnosis, at any stage. Informed consent was obtained from each patient.

The questionnaires were designed based on the previous studies in North America, Canada and Australia, which had looked at general internet cancer related information (Chen and Siu, 2001; Basch *et al*, 2004; Helft *et al*, 2005; Newnham *et al*, 2005, 2006). Modifications were made with additional questions added to meet the aims of this study. As none of the questionnaires from any of the previous studies had been formally validated, an initial validation pilot was conducted in accordance with current recommendations (Sudman and Bradburn, 1982; Oppenheim, 1992; Peterson, 2000; McColl *et al*, 2001). After refinement, the final version was submitted and approved by the institutional Patient Advisory Liaison Services (PALS) and Research and Development Department. The patient questionnaire contained 52 questions, split into six manageable sections. The self-administered questionnaire for HPs contained 28 questions, split into five sections. Questions were predominantly closed and required either simple dichotomous, multiple choice ordinal, or scaled nominal categorical responses.

### Statistical analysis

Statistical analyses were performed using SPSS/Windows version 15.0.1 software (SPSS Inc., Chicago, IL, USA) statistical software. Univariate analyses, Pearson *χ*^2^ test, examined group differences and significant association for patient demographic and characteristic variables, whereas *t*-tests compared means and association of continuous variables. All *P*-values (significance) were two-sided and considered significant if <0.05.

## Results

### Patient questionnaires

In all, 209 (80%) of the 261 eligible patients who received the questionnaires returned them. Five of these questionnaires were incomplete and excluded. Therefore, 204 questionnaires were included in the final analysis. In all, 156 (76%) of the respondents had received online information, of which 104 had specifically received ICI. Of these 104, 73 (70%) conducted online searches themselves and 31 (30%) received the ICI from carers.

Socio-demographic and clinical characteristics for the patient respondents are described in Table 1. The mean age for respondents who used the internet was 54 years (SD=11) and 60 years (SD=9.5) for non-users. The purpose of treatment did not significantly affect the frequency of online searching; ICI was received by 52% of patients undergoing adjuvant or radical chemotherapy compared with 48% of those with palliative intent.

**Figure 1:**
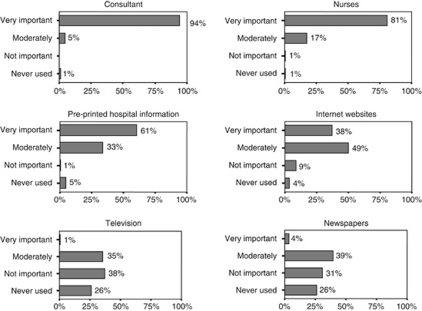
Patient ratings of cancer information resources.

The results show an association between computer access and receipt of ICI; 88% of the 104 respondents who obtained ICI had a home computer whereas the remainder used computers at work or libraries. A total of 79% accessed ICI after discussing chemotherapy with their oncologist who gave them the printed patient information leaflets from macmillan.org.uk. The macmillan.org.uk website was accessed by 48% of the 104 respondents. Other websites that were commonly cited included cancerresearch.org.uk, cancerhelp.org.uk, breastcancercare.org.uk and beatingbowelcancer.org.uk. No other websites were identified by the 104 respondents. Figure 1 illustrates how these respondents rated the importance of ICI, pre-printed hospital information, the media and information obtained from HPs. Telephone help lines, patient support groups, internet chat rooms and other patients with cancer were all considered much less important information resources. Table 2 sets out the impact of ICI on such respondents (including whether they felt anxious, reassured or whether they required further clarification from HPs after reviewing ICI) and identifies key topics that were relevant to their searches (including side effects and how to manage them as well as success rates of chemotherapy).

### HP questionnaires

Questionnaires were given to all 58 HPs at the Centre, of which 47 (81%) were returned completed. In all, 25 (53%) of the HPs were consultant oncologists or specialist registrars and 22 (47%) were specialist oncology nurses. The age of the respondents ranged from 28 to 63 years with a mean age of 43 years (SD=9.9).

All respondents considered that written and verbal patient information supplied by the Centre were essential, whereas 70% considered the internet an important additional information resource. A total of 77% of HPs regularly viewed ICI, in order to be informed when patients asked questions and to consider the accuracy of ICI. The HPs’ opinions of ICI are described in Table 3. A majority of the 47 respondents considered ICI to be inaccurate at times and potentially harmful to patients (in that ICI can add to patient anxiety and be misinterpreted). The HPs’ attitudes towards ICI (including how frequently websites are recommended to patients and ICI content is discussed with them) are shown in Table 4. The HPs’ opinions and perceptions of patients receiving ICI are summarised in Table 5.

## Discussion

### The role of ICI

This study raises interesting issues about the evolving role of the internet in the sphere of patient education and healthcare generally. It is undeniable that reliable information can improve patients’ understanding of chemotherapy and help to allay common concerns. In this study, almost all patient respondents regarded verbal information from HPs and pre-printed leaflets from hospitals as primary sources of information about cancer and its treatment, mirroring the findings of several earlier studies (Meredith *et al*, 1996; Chen and Siu, 2001; Eysenbach, 2003; Balmer, 2005; Helft *et al*, 2005; Newnham *et al*, 2006; James *et al*, 2007; Mayer *et al*, 2007). According to previous studies, additional information sources included discussion with other patients, television, newspapers, and magazines. Telephone helplines and patient support groups were less popular sources of information (James *et al*, 1999; Basch *et al*, 2004; Balmer, 2005). As would be expected with the increased availability of the internet in recent years, the proportion of patients receiving internet information has increased significantly; one would expect this trend to continue. However, given the pressures on health systems worldwide, visits to HPs are often subject to time constraints and patients may not always have the opportunity to raise or discuss all the issues that concern them during the consultation with the HP. In a field as vast, technically challenging and rapidly evolving as oncology, the role of supplementary information outside the traditional consultation setting can be significant, particularly if such information is accessible and suitably targeted at a lay audience. Appropriate written information, including ICI, is useful as it allows the patient to absorb material information at their own pace, refresh key issues and hopefully clarify some of their concerns (Fallowfield *et al*, 1994; Meredith *et al*, 1996; Jenkins *et al*, 2001; Mayer *et al*, 2007). It is not surprising that the majority of patient respondents who received ICI considered it to be an important resource.

### Access to ICI–addressing inequalities

As mentioned above, about three quarters of the patient respondents who accessed ICI searched for such information themselves, whereas the remainder received the information from carers. The results of this study are consistent with previous studies of patients with cancer who searched for health-related information and their socio-demographic characteristics (Chen and Siu, 2001; Eysenbach, 2003; Helft *et al*, 2005; Newnham *et al*, 2006; Mayer *et al*, 2007). Patients who received ICI tended to be younger, better educated with higher household incomes. Accordingly, they were more likely to be familiar with and have regularly used the internet. Of the patients who had not sought ICI, a third cited a lack of internet access, half stated that they were not interested in internet information, whereas the remainder were either not aware of chemotherapy websites or were concerned that they would be unduly anxious after receiving ICI. In relation to the latter point, it should be noted that avoiding or limiting information may be a coping mechanism to decrease distress, manage ambiguity and foster hope (Leydon *et al*, 2000; Mayer *et al*, 2007).

Given the inevitable and growing influence of internet information, inequalities relating to internet access and patients’ internet skills or awareness should perhaps be considered in future health and social policies, in order to ensure that patients across the socio-demographic spectrum have similar, if not equal, opportunities to access appropriate ICI. Improved access and skills relating to the internet cannot, however, be considered in isolation. Future studies and consideration should be given to the following: (1) whether ICI does in fact have a significant and positive impact on patient's well-being; (2) whether the appropriate checks and balances are in place to ensure that patients access ICI that is accurate as opposed to misleading and damaging information; and (3) whether patients and HPs have a consistent understanding of the role of ICI as an educational tool that can be integrated seamlessly into traditional consultation models. Measures may even be introduced to facilitate access to good quality ICI at the cancer centres and other similar settings.

In relation to gender-related inequalities, the proportion of female patients who received ICI was higher than males. This concurs with the results in previous studies where women have been shown to be more active health seekers than men (Fogel *et al*, 2002; Eysenbach, 2003).

There did not appear to be material inequalities in relation to the kinds of cancers researched online; a broad range of cancers seemed to have been looked into by patient respondents. Although the earlier studies have shown an association between cancer type and level of internet usage (Chen and Siu, 2001; Eysenbach, 2003; Helft *et al*, 2005; Newnham *et al*, 2006), the tumour site did not seem to significantly influence whether or not patients received ICI in this particular study.

### Patient perceptions of ICI

The results obtained in this study, particularly in relation to patient perceptions of ICI, can be usefully integrated into future and broader investigations relating to the internet and how it can be meaningfully used to improve the quality of patient care and education, not only in the field of oncology but also in other areas of medicine.

Patient attitudes to ICI were generally positive. Encouragingly, less than a quarter admitted that they were confused by ICI and an even smaller proportion felt anxious having reviewed ICI. This is in contrast with the concerns of the majority of HP respondents who worried about the negative implications of ICI (to be discussed later). Patients frequently fear chemotherapy toxicity, so it was no surprise that the majority searched for ICI relating to side effects, with three quarters wanting to understand the mode of action of chemotherapy, how successfully it controls symptoms and the method of administration. Only half of the patients expressed an interest in new treatments, a third in clinical trials and merely a quarter in alternative treatments. Patients’ hopes are often raised when they learn about a new treatment through the media, but only a third considered that the internet had created unrealistic expectations. More than half felt reassured by ICI, believing that they felt more in control of their treatment, more compliant and better able to cope with the adverse effects. For patients who did not seek ICI, the main reasons, as discussed earlier, were either because they were not interested or had no access to ICI. Only a minority felt that they would be unduly anxious after receiving ICI. This does, however, highlight that patients have different coping styles, which need to be tailored for individually (Miller, 1995).

### The quality and accuracy of ICI–checks and balances

Given that patients do value ICI as a useful source of information (and the role of ICI is likely to grow with the advent of the information age), it has become increasingly important to ensure that patients are able to access good quality and accurate information. This consideration applies equally across all aspects of medicine and healthcare. In this regard, the following considerations are crucial: (1) patients must be given some guidance and direction to websites that are accurate, reliable and user-friendly (in that the content must be easily understood by the lay person and not be overwhelming, confusing or misleading); (2) patients must be given the opportunity to clarify any doubts or to raise questions following their review of ICI; and (3) the quality of recommended websites must be maintained and be subject to ongoing review and validation by HPs.

(2) is an important consideration and HPs must be live to the fact that patients will become increasingly able to obtain information about cancer outside the consultation and will have follow-on questions and concerns that they may wish to raise with HPs. Patients must also be made to understand the time and other constraints of a consultation and appreciate that ICI can only supplement or complement case-specific advice given by the HP.

In relation to (3), it is reassuring that the vast majority of patient respondents in this study listed peer-reviewed websites such as macmillan.org.uk or cancerresearchuk.org as their source of ICI. Although almost half of the study patients thought they were able to identify which websites they considered to be trustworthy, more than half indicated that they required further guidance from HPs. This is something that should be addressed in future.

### HP perceptions of ICI

Although earlier studies have described HPs’ concerns that the internet can be inaccurate, distressing or overrated by patients, it was encouraging that most HPs in this study believed that the internet has the potential to increase patients’ understanding of the disease and its treatment (Biermann *et al*, 1999; Chen and Siu, 2001; Helft *et al*, 2003; Newnham *et al*, 2005). Although two thirds had a generally supportive attitude, the majority considered that the internet could be detrimental and cause harm to more vulnerable patients. There were concerns that patients may develop unrealistic expectations, become anxious or confused, as a result of their inability to interpret and assess the quality, accuracy and reliability of information. Almost three quarters thought that patients may request unproven treatments, whereas around half feared that a new or alternative treatment may be sought, which was either unavailable due to high costs, or be unsuitable for the patients’ condition. Two thirds believed patients were interested in prognosis following chemotherapy but less than half considered their interest in side effects. Most HPs thought that patients search to seek reassurance and a better understanding of chemotherapy, or are hopeful to find a successful treatment for their cancer.

The sharing of ICI with HPs provides an opportunity for the patient to clarify any confusion or misunderstandings. Despite more than half of the patients indicating that they would have liked to discuss their findings, the majority had not been given an opportunity. The HPs not only underestimated the proportion of patients who received internet information, but some appeared unaware of the patients’ needs for guidance and opportunities for discussion. Only a small proportion of HPs recommended websites unless specifically requested by patients. It should perhaps be considered important for HPs to determine what information patients are most interested in, how they can offer guidance to credible websites, and to realise that patients may need assistance to accurately interpret the information. Although some found it difficult to allow time, most recognised that this is not a challenge to their authority but an effort by patients to learn more and better understand their disease and chemotherapy treatment. This raises questions as to whether there is a perceived or real need to actively offer patients the opportunity to discuss retrieved information, as discrepancy of information from HPs compared with ICI may raise issues from patients. Discussion of ICI could in fact be an opportunity to strengthen this relationship by clarifying concerns, relieving anxiety and assisting in more complex decision making. In addition to offering guidance to patients, it should be acknowledged that some will prefer not to receive ICI, and they should be reassured that HPs provide all the information required for the treatment.

As the availability of ICI continues to develop, HPs should be involved in its regulation and peer review. This will ensure that information is evidence based and presented in a balanced format suitable to the patients’ level of understanding while maintaining homogeneity of websites’ standards. A recently developed web-based tool in the UK is the National Cancer Action Team's ‘Patient Information Prescription’ (Cancer Patient Information Pathways, 2011). This offers a consistent approach to disease and treatment information, enabling HPs to provide standardised peer reviewed information leaflets, and direct patients to quality-reviewed websites according to individual needs. Perhaps the best application of ICI should be for it to be integrated with current traditional consultation models to enhance patient experience and provide additional information in conjunction with that provided by HPs.

In conclusion, although the primary source of chemotherapy information remains that of HPs, this study has shown that ICI is generally perceived by patients to be a valuable information source, which is used to augment information traditionally obtained through HPs. Although HPs had some understandable concerns regarding the possible detrimental effect to patients and their ability to interpret internet information, the majority generally recognised and supported the need for patients to retrieve internet information to improve their understanding of chemotherapy treatment. It has nevertheless revealed discrepancies that exist between HPs perception and patients’ needs with regards to ICI seeking behaviour. This emphasises a need for HPs to work more closely with patients in addressing their concerns and directing them to credible websites.

This study has therefore highlighted the need to: (1) improve access to ICI; (2) to reassess current consultation models to address individual needs; (3) to provide guidance and; (4) to maintain quality assurance of accredited chemotherapy websites. It follows that the potential exists to integrate ICI with current traditional consultation models synergistically in order to enhance patient experience.

## Figures and Tables

**Table 1:** Patient socio-demographics characteristics

**Variable**	**Total (*n*=204) count (%)** a	**Chemotherapy internet user (*n*=104) count (%)** b	**Chemotherapy internet non-user (*n*=100) count (%)** b	***χ*^2^**-**test**	***P*-value**
*Gender*					
Male	76 (37)	30 (40)	46 (60)	5.71	0.017
Female	128 (63)	74 (58)	54 (42)		
					
*Patient's age (years)*					
⩽40	19 (9)	15 (79)	4 (21)	19.09	0.001
41–50	30 (15)	20 (67)	10 (33)		
51–60	72 (35)	40 (56)	32 (44)		
61–70	63 (31)	24 (38)	39 (62)		
⩾71	20 (10)	5 (25)	15 (75)		
					
*Annual household income*					
<£12 000	52 (26)	11 (21)	41 (79)	38.18	<0.001
£12 000–£20 000	46 (22)	22 (48)	24 (52)		
£21 000–£30 000	47 (23)	24 (51)	23 (49)		
£31 000–£40 000	32 (16)	25 (78)	7 (22)		
>£40 000	27 (13)	22 (82)	5 (18)		
					
*Qualifications*					
None	71 (35)	26 (37)	45 (63)	10.73	0.004
GCSE/O-levels	78 (38)	42 (54)	36 (46)		
HNC/diploma /degree	55 (27)	36 (66)	19 (34)		
					
*Occupation (current or previous)*					
Housewife	31 (15)	13 (42)	18 (58)	17.78	0.001
Manual worker/tradesman	49 (29)	20 (34)	39 (66)		
Clerical/sales assistant	53 (26)	28 (53)	25 (47)		
Teaching/health professional	25 (12)	19 (76)	6 (24)		
Management	36 (18)	24 (67)	12 (33)		
					
*Tumour site*					
Breast	59 (29)	39 (66)	20 (34)	10.93	0.067
Gynaecological	22 (11)	11 (50)	11 (50)		
Lung	24 (12)	8 (33)	16 (67)		
Lower gastrointestinal	58 (28)	27 (47)	31 (53)		
Upper gastrointestinal	23 (11)	9 (39)	14 (61)		
Head and neck, sarcoma, urology	18 (9)	10 (56)	8 (44)		

a % Within the total.

b % Within the categorical variable.

**Table 2:** Patients key interests and opinions of ICI

**Variable**	**Total (*n*=104) count (%)**
*Key interests of patients*	
List of side effects	86 (83)
Why side effects occur	80 (77)
How side effects are managed	75 (72)
Mode of action of chemotherapy	88 (85)
How chemotherapy is administered	72 (69)
Chemotherapy treatment success	65 (63)
New treatments	58 (56)
Clinical trials	36 (35)
Alternative treatments	28 (27)
	
*How helpful was internet information*	
Very helpful	57 (55)
Moderately helpful	47 (45)
	
*Internet answer additional questions*	
All	8 (8)
Some	72 (69)
None	24 (23)
	
*Internet clarified hospital information*	
Yes	63 (61)
No	41 (39)
	
*Internet information reassured patient*	
Yes	57 (55)
No	47 (45)
	
*Patient felt confused*	
Yes	16 (15)
No	88 (85)
	
*Patient felt anxious*	
Yes	11 (11)
No	93 (89)
	
*Patient coped better with treatment*	
Yes	55 (53)
No	49 (47)
	
*Created realistic hope or expectations*	
Yes	64 (62)
No	40 (38)
	
*Knew which websites were trustworthy*	
Yes	48 (46)
No	56 (54)
	
*Required guidance to websites*	
Yes	61 (59)
No	43 (41)
	
*Wanted to discuss with health professional*	
Yes	64 (61)
No	40 (39)
	
*Opportunity to discuss with health professional*	
Yes	63 (61)
No	41 (39)

Abbreviation: ICI, internet chemotherapy information.

**Table 3:** Health professionals’ opinions of ICI

**Variable**	**Total (*n*=47) count (%)**
*How often is ICI accurate*	
Sometimes	40 (85)
Rarely	7 (15)
	
*Can the internet cause harm to the patient*	
Yes	41 (87)
No	6 (13)
	
*Mechanisms of harm to patient*	
Misinterpretation of internet information	39 (83)^a^
Create unrealistic patient expectations	36 (77)^a^
Causes anxiety, distress or confusion	37 (79)^a^
Unable to assess quality/reliability	35 (75)^a^
Information is inaccurate or poor quality	28 (60)^a^
Unproven/alternative treatment requested	33 (70)^a^
Treatment is unavailable due to cost	24 (51)^a^

Abbreviation: ICI, internet chemotherapy information.

a Total does not add up to total number, as none, or more than one question could be answered in this section.

**Table 4:** Health professionals′ recommendation of websites and discussion of ICI with patients

**Variable**	**Total (*n*=47) count (%)**
*HPs′ estimation of patients who received ICI*	
25% of patients	27 (57)
50% of patients	16 (34)
75% of patients	4 (9)
	
*Do HPs routinely recommend websites to patients*	
Sometimes	9 (19)
Rarely	38 (81)
	
*If patient requests ICI, are websites recommended*	
Often	36 (77)
Sometimes	7 (15)
Rarely	4 (8)
	
*HPs′ estimation of patients who want to discuss ICI*	
25% of patients	32 (68)
50% of patients	12 (26)
75% of patients	3 (6)
	
*How often do HPs discuss ICI*	
Always	9 (19)
Most of the time	15 (32)
Sometimes	17 (36)
Rarely	6 (13)

Abbreviations: HPs, health professionals; ICI, internet chemotherapy information.

**Table 5:** Health professionals’ opinions and perceptions of patients receiving ICI

**Variable**	**Total (*n*=47) count (%)**
*Attitude towards patients who searched for ICI*	
Supportive	32 (68)
Neutral	15 (32)
	
*Do HPs feel challenged when patients discuss ICI*	
Sometimes	5 (10)
Rarely	22 (47)
Never	20 (43)
	
*Why do patients search for ICI*	
Look for complimentary/alternative treatments	44 (94)^a^
Seek reassurance/understanding of treatment	37 (79)^a^
Hope to find a new treatment	35 (75)^a^
Learn about prognosis after chemotherapy	29 (62)^a^
Look for the information on the side effects	23 (49)^a^
Insufficient information supplied by Oncology Center	9 (19)^a^
	
*How often do patients accurately interpret ICI*	
Often	9 (19)
Sometimes	35 (75)
Rarely	3 (6)
	
*Are patients who search the internet better informed*	
Better	19 (40)
No difference	28 (60)
	
*Do internet searchers cope better or worse*	
No difference	39 (83)
Worse	8 (17)

Abbreviations: HPs, health professionals; ICI, internet chemotherapy information.

a Total does not add up to total number, as none, or more than one question could be answered in this section.
